# Change of Flavonoid Content in Wheatgrass in a Historic Collection of Wheat Cultivars

**DOI:** 10.3390/antiox13080899

**Published:** 2024-07-25

**Authors:** Chu-Yang Wang, Xiao-Ming Li, Han-Xiao Du, Yan Yan, Zhong-Zhong Chen, Chen-Xi Zhang, Xin-Bo Yan, Shui-Yuan Hao, Jin-Ying Gou

**Affiliations:** 1 MOE Engineering Research Center of Gene Technology, Institute of Plant Biology, School of Life Sciences, Fudan University, Shanghai 200438, China; 19110700147@fudan.edu.cn (C.-Y.W.); 18110700005@fudan.edu.cn (X.-M.L.); as00419@163.com (H.-X.D.); 17110700056@fudan.edu.cn (Y.Y.); 14210700019@fudan.edu.cn (Z.-Z.C.); 2 Xianghu Laboratory, Hangzhou 311231, China; 3 Frontiers Science Center for Molecular Design Breeding, China Agricultural University, Beijing 100193, China; 2022301010125@cau.edu.cn (C.-X.Z.); ysdj@cau.edu.cn (X.-B.Y.); 4 Department of Agronomy, Hetao College, Bayannur 015000, China

**Keywords:** wheatgrass, antioxidant activity, flavonoids, powdery mildew

## Abstract

Wheatgrass is recognized for its nutritional and medicinal properties, partly attributed to its flavonoid content. The objective of this study was to assess the flavonoid content and antioxidant properties of wheatgrass obtained from a wide range of 145 wheat cultivars, which included Chinese landraces (CL), modern Chinese cultivars (MCC), and introduced modern cultivars (IMC). The flavonoids were extracted using a solution of 80% methanol, and their content was evaluated using ultra-high-performance liquid chromatography-mass spectrometry (UHPLC-MS). The results revealed the assessed cultivars showed significant variation in their total flavonoid content (TFC), with MCCs generally having higher amounts compared to CLs. PCA analysis demonstrated clear variations in flavonoid profiles between different cultivar groups, emphasizing the evolutionary inconsistencies in wheat breeding. The antioxidant assays, ABTS, DPPH, and FRAP, exhibited robust abilities for eliminating radicals, which were found to be directly associated with the amounts of flavonoids. In addition, this study investigated the correlation between the content of flavonoids and the ability to resist powdery mildew in a collection of mutated wheat plants. Mutants exhibiting heightened flavonoid accumulation demonstrated a decreased severity of powdery mildew, suggesting that flavonoids play a protective role against fungal infections. The results highlight the potential of wheatgrass as a valuable source of flavonoids that have antioxidant and protective effects. This potential is influenced by the genetic diversity and breeding history of wheatgrass. Gaining insight into these connections can guide future wheat breeding endeavors aimed at improving nutritional value and in strengthening disease resistance. The current finding provides critical information for developing wheatgrass with high flavonoid content and antioxidant activity.

## 1. Introduction

Aging is a significant global challenge, marked by sharp increases in chronic diseases, such as diabetes, colon cancer, and Alzheimer’s disease. In addition, a poor diet contributes to a younger age at which aging manifests itself and increases its incidence. Chronic disease prevalence lowers quality of life and puts extra burden on public health system. Use of phytonutrients can be instrumental to address this issue. Studies reported the free radical-scavenging abilities of flavonoids, given the pivotal role of reactive oxygen species (ROS) in developing chronic diseases. Flavonoids have demonstrated potent antioxidant properties in laboratory settings [1].

Flavonoids prevent age-related declines in memory and significantly promote human cognitive function [2]. Evidence suggests that luteolin exhibits therapeutic potential in a transgenic *Drosophila melanogaster* model of Alzheimer’s disease [3]. Apigenin can mitigate the lifespan-reducing effects of D-galactose by enhancing antioxidant capabilities and preventing mitochondria-driven apoptosis in *Drosophila* [4]. To reduce the incidence of chronic diseases, the preventive effect of a daily diet is better than medical intervention. To minimize the incidence of chronic diseases, the preventive effect of a daily diet is better than medical intervention. Therefore, the concept of “Nutrition-sensitive agriculture (NSA)” was proposed in the Second International Conference on Nutrition (ICN2) in 2014 to address the increasingly severe problem of chronic diseases.

Due to its antioxidant activity, wheatgrass possesses preventive and therapeutic effects on various chronic diseases. In their early stages, the wheatgrass contained flavone-C-glycosides such as isoorientin and isovitexin [5]. Another study identified 13 flavonoid glycosides in wheatgrass, which supports the above finding [6]. The aglycones of flavonoid glycosides are mainly luteolin, chrysin, and apigenin; however, the structure of the specific glycosylation modification is complex and unclear. Moreover, the isolation and purification of the flavonoids in wheatgrass revealed disaccharide modifications and bis-glycosylation modifications on these glycosides, in addition to monoglycosylation modification [7]. However, recent studies have primarily focused on identifying compound components, highlighting the need for broader screening of germplasm resources to support the practical application of wheatgrass.

Flavonoids play a role in plants’ defense response against pathogens. Wheat production faces significant losses due to various fungi, such as powdery mildew (caused by *Blumeria graminis* f. sp. *Tritici*, *Bgt*) [8]. Plant antimicrobial agents are mainly secondary metabolites. Soluble phenylpropanoids participated in the defensive responses against a soil-borne vascular pathogen *Verticillium longisporum* in Arabidopsis [9]. The introduction of the wheat *Lr34* gene into sorghum (*Sorghum bicolor*) increased the expression levels of multiple genes involved in phenylpropanoid synthesis, such as *SbFNSII*, *SbFNR*, and *SbDFR3*. The above change corresponded with the higher levels of luteolinidin observed in genotypes carrying *Lr34res* at 72 h post-inoculation (hpi) [10]. The significant presence of flavonoid glycosides in barley (*Hordeum vulgare* L.) strengthens cell walls to confine *Fusarium graminearum* in the initial infection area [11]. Exogenous kaempferide and apigenin application on wheat spikes increased wheat resistance to Fusarium head blight (FHB) [12].

Screening and analyzing the germplasm and commercial cultivars of wheat is an important step for identifying genotypes with high flavonoid and antioxidant properties. The objective of this study is to evaluate the changes in flavonoid content in a historical collection of wheat cultivars. By identifying genotypes with high flavonoid content, we aim to select potential parents for breeding programs to develop elite wheat cultivars with enhanced antioxidant properties.

## 2. Materials and Methods

### 2.1. Reagents and Chemicals

Acetonitrile (HPLC-grade) and Methanol (HPLC-grade) were procured from Sigma-Aldrich (St. Louis, MO, USA). Standards of isoorientin, isovitexin, and luteolin were obtained from Shanghai Yuanye Biotechnology Co., Ltd. (Shanghai, China). Sodium nitrite, potassium persulfate, aluminum chloride, and ferric chloride were obtained from Sangon Biotechnology Co., Ltd. (Shanghai, China). ABTS (2,2′-Azino-bis (3-ethylbenzothiazoline-6-sulfonic acid)), TPTZ (2,4,6-Tripyridyl-S-triazine), and DPPH (1,1-Diphenyl-2-Picrylhydrazyl) were products obtained from Ourchem (Sinopharm Chemical Reagent, Shanghai, China).

### 2.2. Plant Genetic Resources and Growth Conditions

The wheat varieties were all previously reported [13]. The germination and growth conditions for the wheat seedlings were as follows: immerse the dried wheat seeds in tap water for 16 h, sew them on pre-soaked filter paper, and maintain them at room temperature for 1 to 2 days to allow for germination. Upon observing the emergence of white embryonic roots, the germinating seeds were transferred to a 4 °C refrigerator and stored for a week before being transferred to the sterilized culture soil. The seedlings were grown in a chamber set at 22 °C under a 16 h light/8 h dark cycle. After one week, the aerial parts of the seedlings were collected, quickly washed with tap water, and then utilized for flavonoid extraction.

### 2.3. Extraction of Flavonoids from Wheatgrass

The samples were frozen in liquid nitrogen and homogenized in a tissue grinder, set at 55 Hz for 30 s, and repeated twice to ensure thorough homogenization. In total, 100 mg of the resulting fine powder was transferred into a clean centrifugal tube and received 80% methanol at a ratio of 1 mg of plant material to 10 μL of solvent. The samples were mixed in the tissue grinder at 60 Hz for one minute, repeating the process twice. The samples were sonicated for 30 min to efficiently extract the flavonoids from the leaf tissue. The homogenate was centrifuged at 12,000 rpm for 15 min. The supernatant was carefully drained into a fresh centrifuge tube and stored at 4 °C overnight. The next day, the supernatant was centrifuged at 12,000 rpm for 15 min. The clear supernatant was transferred to a new 1.5 mL centrifuge tube. The resulting solution was either used immediately for analysis or was stored in a low-temperature refrigerator at −80 °C for short-term storage. In the field test, the EMS mutants and control plants were grown without pesticide application at the farm in Fudan University, Shanghai, China (N: 31.329586, E: 121.522701). The powdery mildew naturally occurred in the field.

### 2.4. Total Flavonoid Content (TFC) Detection in Wheatgrass

We first added 90 μL of a 30% methanol solution into each well of a 96-well microplate and then mixed them with 10 μL flavonoid extract. The mixture was allowed to incubate at room temperature for 5 min to ensure proper blending. Following the initial incubation, each well was added 6 μL of a 5% sodium nitrate (NaNO_3_) solution, and the palter was rested for another 5 min. Subsequently, each well was received 6 μL of 5% aluminum chloride (AlCl_3_) solution and kept undisturbed for 10 min to facilitate the formation of the flavonoid–aluminum complex. Afterward, each well received 50 μL of 1 M sodium hydroxide (NaOH) solution to terminate the reaction. The plate was then set aside to rest for 5 min to stabilize the color change. Once the reaction was complete, the absorbance was measured in a microplate reader, using authentic catechin as the standard.

### 2.5. Antioxidant Activity Analysis

The 1,1-Diphenyl-2-picrylhydrazyl radical (DPPH) was dissolved into 80% methanol at 10 mM to prepare a stock solution, which was diluted with 80% methanol until the OD_517_ equaled 0.5 to prepare the DPPH working solution. In total, 10 μL of samples (standard, soluble, and wall-bound phenolic) were mixed with 190 μL of DPPH working solution in the dark. The samples were shaken for 10 s every 5 min at RT for 30 min, and we recorded their absorbances at 517 nm to calculate the corresponding DPPH radical scavenging activity following the standard curve ranging from 50 to 600 µM of luteolin. We used 80% methanol as the negative control.

A colorimetric method was applied to quantify the 2,2′-azino-bis (3-ethylbenzothiazoline-6-sulfonic acid) (ABTS)-removing activity. The ABTS reagent (7 mM ABTS and 2.45 mM K_2_S_2_O_8_, both in 5 mM PBS, pH 7.4) was prepared in the dark overnight, and was diluted with 5 mM of phosphate buffer (PBS) to OD_734_ equaling 0.7 ± 0.02. A volume of 195 μL ABTS reagent and 5 μL of crude extracts was mixed in a transparent 96-well plate and kept at 37 °C for five minutes. The absorbance at 734 nm was quantified using 80% methanol as the blank and PBS as the negative control. Authentic luteolin ranging from 50 to 600 µM worked as the standard to build the standard curve.

The Ferric ion-reducing antioxidant power (FRAP) working solution contained 300 mM pH3.6 acetate buffer, 10 mM TPTZ (TPTZ, 2,4,6-Tris (2-pyridyl)-s-triazine), and 20 mM FeCl_3_ at a ratio of 10:1:1 (*v*/*v*). The FRAP solution (190 μL) and 10 μL of the samples (standard, soluble, and wall-bound phenolic) were mixed in a transparent 96-well plate and kept at 37 °C in the dark for 30 min with a 10 s shake every 5 min. The absorbance at 593 nm was measured to calculate the FRAP radical-scavenging activity according to the standard curve ranging from 50 to 600 µM with 80% methanol as the negative control [14].

### 2.6. Characterization of Flavonoids

The samples were injected into a column at 30 °C, with the flow rate at 0.8 mL/min and the detection wavelength at 340 nm. The UHPLC-MS system included an Agilent 1290 ultrahigh-performance liquid chromatography system (UHPLC) coupled with an Agilent 6530B Accurate-Mass q-TOF (UHPLC-qTOF-MS) (Agilent Technologies, Inc., Lexington, MA, USA). Mass spectrometers were operated in the positive ion mode (ESI+). The MS spectra were acquired with a spectral mass-to-charge ratio range (*m*/*z*) of 110–950. All of the MS spectra were processed using the software package Qualitative Analysis and Profinder (Ver 8.08.00, Agilent Technologies, Inc., Lexington, MA, USA).

### 2.7. Detection of Flavonoids in Wheatgrass

The chromatographic column model is Thermo Accucore C18 (2.1 × 100 mm, 2.6 μm), employing a gradient elution method (Table 1) equipped with the Thermo UltiMate 3000 Instrument (Thermo Fisher Scientific, Dreieich, Germany). The sample injection volume is 10 μL, the column temperature is set at 30 °C, and the flow rate is 0.8 mL/min. A DAD (Diode-Array Detector) is used for detection with the wavelength set to 340 nm. Chromatographic data are processed using the Thermo Chromeleon 7.2 SR4 software.

### 2.8. Powdery Mildew Infection Analysis

In the field trial at Fudan University in Shanghai, the EMS mutants in the Kronos background were infected by naturally occurring powdery mildew pathogens [5]. Flag leaves at the ‘milk-ripe’ stage (Feekes Scale11.1) were collected and freeze-dried for further analysis. In controlled lab conditions, seedlings at the two leaves and one heart stage were inoculated with an equal density of fresh spores of an isolated *Bgt E09* pathogen [15]. The samples were pictured and stained with Coomassie bright-blue (CBB) staining buffer at 4 or 8 days post-inoculation (DPI) to show powdery mildew development. At 8 DPI, the samples were collected to extract DNA and quantify the pathogen biomass.

### 2.9. DNA Extraction and Quantitative Real-Time PCR

Total DNA was extracted from 20 mg of the leaf powder prepared above using 1.0 mL of extraction buffer (60 °C) to each tube. The tubes were mixed well, incubated in a 60 °C water bath for 30 min, and mixed every 10 min. After cooling down to RT, the samples were mixed with 300 μL of ammonium acetate (stored at 4 °C) and incubated in a 4 °C refrigerator for 15 min. After centrifugation for 15 min at 13,000 rpm at 4 °C, supernatants were carefully drained into a new 1.5 mL tube with 360 μL of isopropanol. DNA was pelleted after centrifugation for 15 min at 13,000 rpm at 4 °C, rinsed with 75% ethanol, and air-dried. The dried DNA was dissolved in 100 μL of ddH_2_O overnight in a 4 °C refrigerator, and then was analyzed using real-time PCR using HieffTM qPCR SYBRTM Green Master Mix (Shanghai Yeasen Biotechnology, Shanghai, China) in a CFX 96-Realtime system (BIORAD, Singapore). Wheat or powdery mildew actin genes were applied with the following primers: TaAct-F(5′ TGTTGGTGATGAGGCGCAGT 3′) and TaAct-R (5′ TGCGACGTACATGGCAGGAA 3′), or BgtAct-F (5′ TTTGACCAGGAAATACAGACC 3′) and BgtAct-R (5′ AGAGCCACCAATCCATACAG 3′), respectively. Real-time PCR was performed following the PCR master mix instruction manual.

## 3. Results

### 3.1. The Flavonoids and Antioxidant Capacities in Wheatgrass of a Historical Population

**Figure 1:**
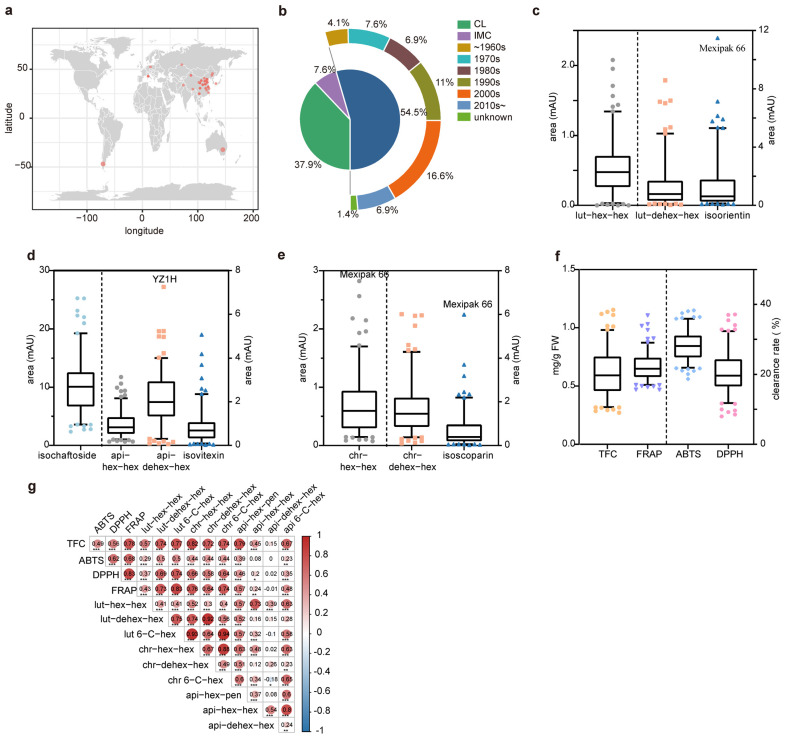
The geographic distribution and flavonoids of the 145 wheat varieties. (**a**) According to their geographic distribution and breeding period, the varieties are categorized into the Chinese landraces (CL), introduced modern cultivars (IMC), and modern Chinese cultivars (MCC) groups. (**b**) The MCC group is further subdivided into six subgroups, each representing a decade. (**c**) The content of three luteolin glycosides. (**d**) The content of four types of apigenin glycosides. (**e**) The content of three types of chrysoeriol glycosides. (**f**) The content of the total flavonoid content and the detection of three types of free radical scavenging capabilities. The box plots to the left of the dashed line use the horizontal axis on the left, while those to the proper use the horizontal axis on the right. The data points represent the top and bottom 5% of the 145 materials. (**g**) Correlation analysis between various flavonoids and antioxidant potentials in 145 samples. The black numbers represent the two indicators’ correlation coefficient (*r*-value). The size and color of the circles can also be used to compare the *r*-values, with red indicating a positive correlation and blue indicating a negative correlation. The darker the color, the larger the absolute value of the *r*-value. The asterisks denote the significance level of the correlation analysis, as follows: * *p* < 0.05, ** *p* < 0.01, and *** *p* < 0.0001.

A total of 145 wheat cultivars were collected, comprising 55 Chinese landraces (CL), 79 modern Chinese cultivars (MCC), and 11 introduced modern cultivars (IMC). Neuzucht was introduced from Germany; a vital founder parent, Lumai1Hao, was bred from this. Mexipak 66 was a landmark Green-Revolution cultivar from CIMMYT, and Orofen was an international line that worked as import founder parents in Chinese wheat-breeding history (Figure 1a, Table S1).

According to the pattern of wheat varieties in China bred every ten years, we further subdivided 79 modern Chinese cultivars into six subgroups (Figure 1b). Among them, 34 varieties were bred after 2000, including the mainly popularized varieties or founder parents JiMai22 (JM22), Bonong Aikang58 (AK58), etc. Therefore, the flavonoid content in the wheat leaves of this group can reflect the content of recent wheat varieties in China (Table S1). Principal component analysis (PCA) and evolutionary tree analysis based on the resequencing data showed significant differences between CL and the other two groups (Figure S1a,b), which is consistent with the other literature reports [13].

The total flavonoid content (TFC) among the above 145 wheat cultivars varies significantly, with a coefficient of variation of about 23% (Figure 1c–e). The highest and lowest content disparity is as much as four times (Figure 1c–e). The major flavonoid glycosides were flavonoid glycosides (Peak1, Peak3, and Peak4), apigenin flavonoid glycosides (Peak2, Peak5, Peak7, and Peak9), and chrysoeriol flavonoid glycosides (Peak6, Peak8, and Peak10) based on their aglycones (Figure S2). Their glycosylation modification had a clear trend, as follows: Peak4, Peak9, and Peak10 are all 6-C-glycosylation, while Peak2 (Isochaftoside) is a disaccharylated glycoside, where the sixth and eighth carbon of apigenin are modified by arabinose and glucose, respectively (Table S2). Mexipak 66 has a higher content of isoorientin, isoscoparin, and isoscoparin-2-O-glucoside content (Figure 1c and Figure S2), and YanZhan1 has the highest content of Peak 7 (Figure 1d). More changes were observed in the amounts of luteolin flavonoid glycosides and chrysoeriol flavonoid glycosides than in the amounts of apigenin flavonoid glycosides. For example, the coefficient of variation of isoorinin (peak4) was as high as 128% (Figure 1c), and that of isoscoparin (peak10) was 114% (Figure 1e).

The free radical scavenging activities, ABTS, DPPH, and FRAP, of these 145 wheat varieties change accordingly with the flavonoid content (Figure 1f). Overall, the content of luteolin flavonoids exhibited a stronger correlation than those of apigenin flavonoids (Figure 1g). There were significant correlations between the content of flavonoid glycosides with the same glycosylation modification patterns: the correlation coefficient between isoorientin (peak4) and isoscoparin (peak10) was as high as 0.94, and the correlation coefficient between peak3 and peak8 also reached 0.92, which may be caused by the fact that C-glycosylation modification occurs firstly under the catalysis of F2H and CGT then under the closed-loop to generate stable flavonoid glycosides [16].

### 3.2. Flavonoid Content in Young Leaves of MCC Group Were Higher Than Those in CL Group

**Figure 2:**
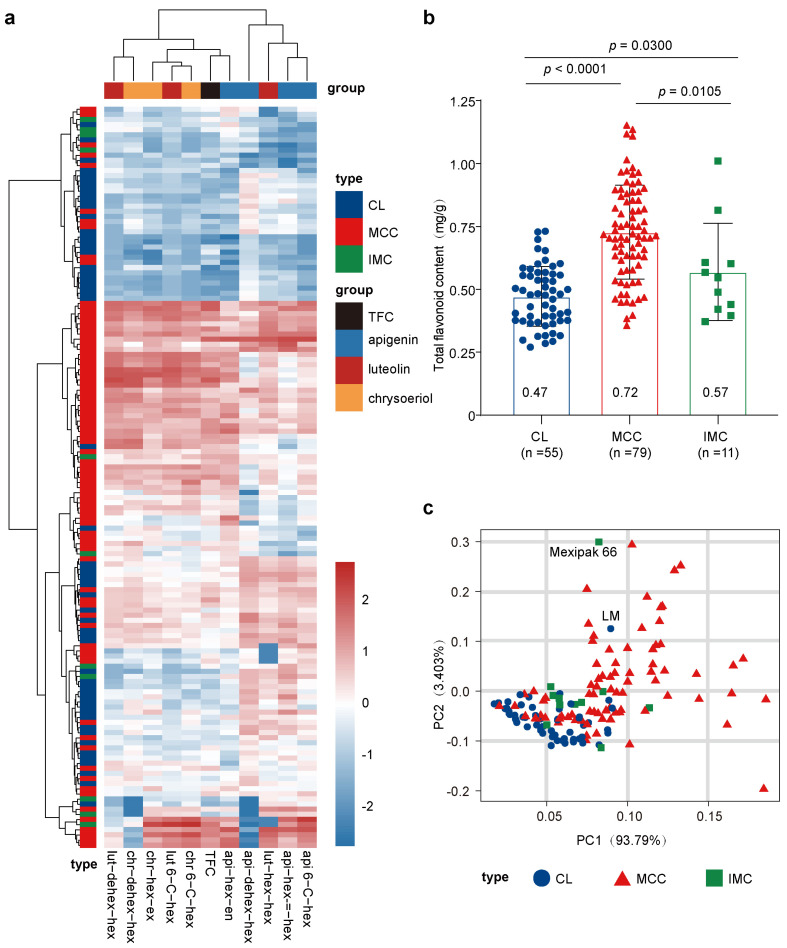
Comparison of flavonoids and antioxidant capabilities among three wheat groups. (**a**) Heatmap analysis of flavonoids and antioxidant capabilities in 145 wheat samples, with raw data normalized using log_2_ (detected value/average value), and clustering analysis performed on both row and column data to facilitate interpretation. (**b**) Total flavonoid content in the young leaves of the three groups, with the numbers in the bar chart representing the mean total flavonoid content in the young leaves of wheat for the CL group (*n* = 55), MCC group (*n* = 79), and IMC group (*n* = 11) individuals. A Student’s *t*-test was conducted between each pair of groups, and the *p*-values are indicated above the graph. (**c**) Principal component analysis (PCA) of the total flavonoids and the content of 10 flavonoid glycosides in the 145 wheat samples, with the first principal component accounting for 93.79% of the contribution, and the second principal component accounting for 3.403% of the contribution. Samples from the CL group are represented by blue circles, those from the MCC group by red triangles, and those from the IMC group by green squares.

Having investigated the variations of flavonoids in the whole population, we then compared the flavonoid content in young leaves among different subgroups. A clustering analysis based on the flavonoid glycoside content classified the 145 cultivars into different groups representing their differences in flavonoid composition (Figure 2a). Three luteolin flavonoids and two chrysoeriol flavonoids formed a cluster of chemicals, which was consistent with the results of correlation analysis, indicating that the accumulation of these flavonoid glycosides had a direct impact on the accumulation of total flavonoid content in young leaves (Figure 2a).

In the clustering by row, the total flavonoid content of most MCC varieties were mainly higher than that of the CL group (Figure 2a,b). More importantly, we noticed that some MCC varieties and CL varieties clustered together, suggesting that there were also differences in flavonoid content in young leaves of wheat within the MCC group. The PCA of flavonoid glycosides showed differences in flavonoid glycoside content in young leaves with MCC wheat varieties. Except for Mexipak 66 and Laomai, most CL and IMC are clustered in the lower left corner. MCC can be roughly divided into the following two types: one is close to the CL group, and the other is far away (Figure 2c). The MCC wheat cultivars close to CL have a lower flavonoid glycoside content. Further subgrouping of the MCC revealed that materials with similar flavonoid content to the CL group were mostly those bred in an earlier stage (before the 1980s), while MCCs differed significantly from the CL group were mostly varieties bred later, and usually had more flavonoids (Figure S3).

### 3.3. The Trend in Flavonoid Content in MCCs over the Breeding Process

**Figure 3:**
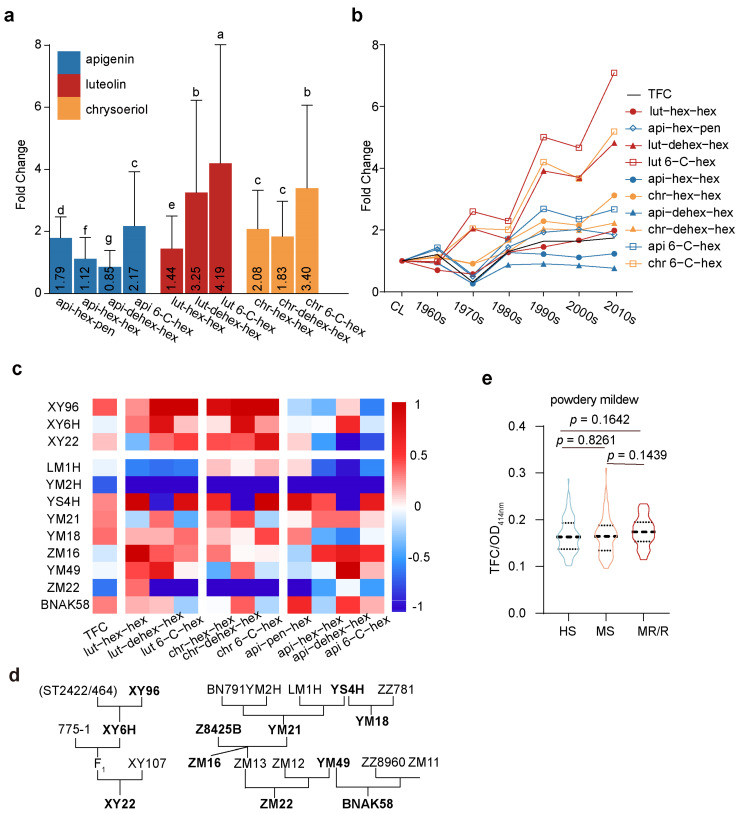
Progressive accumulation of flavonoid content in Chinese wheat varieties over breeding eras. (**a**) Take the average values of the CL group as a baseline; the MCC group is normalized to calculate foldchange relative to CL. Paired *t*-tests were conducted within the ten flavonoid glycosides. Black numbers on the bar chart indicate the average fold change. The letters, a to g, indicate different groups in statistical analysis. (**b**) The increase in flavonoid content in wheat varieties developed during different eras in MCC relative to the CL group, with blue representing apigenin glycosides, red representing luteolin glycosides, and orange representing chrysoeriol glycosides. The shapes are used to differentiate flavonoids with distinct glycosylation modifications. (**c**) Flavonoid content in the young leaves of wheat core parental lines in MCC. (**d**) The breeding information for certain founder parent lines from Shaanxi and Henan provinces. (**e**) The flavonoid content in cultivars with different powdery mildew resistance. The varieties tested in this experiment are highlighted in bold black font.

To verify the hypothesis proposed previously, we first compared the content of different flavonoid glycosides in the MCC group with that in the CL group, and conducted a paired *t*-test on the data of each group. According to the classification of aglycones, the content of luteolin flavonoids showed the greatest difference between the two groups. Among them, the increase in isoorientin was as much as four times, significantly higher than the other nine flavonoid glycosides. The content of chrysoeriol flavonoid glycosides also increased by 1.8–3.4 times, significantly higher than the other three apigenin flavonoid glycosides (Figure 3a).

The distribution pattern of the increase in flavonoid glycoside content also appears to be related to the types of glycosylation modification. Among flavonoid glycosides with the same aglycone, those with 6-C glycosylation modification exhibited high content, and the foldchange was significantly higher than that of other flavonoid glycosides, more than twice in all three 6-C glycosylation-modified flavonoids (Figure 3a). Subsequently, we divided 79 varieties into six groups, each spanning a decade, and it was evident that the total flavonoid content in wheat seedlings has been continuously increasing with the development of wheat breeding. The growth rate in the first half of the century was relatively rapid, increasing from 0.47 mg/g in the early CL group to 0.77 mg/g in the 1990s, after which the growth rate slowed (Figure 3b).

Among the ten flavonoid glycosides, isoorientin content increased the fastest, with varieties bred in the 2010s having nearly eight times the isoorientin content of the CL group (Figure 3b); Isoscoparin followed closely behind. In contrast, the content of the two apigenin flavonoid glycosides (api-hex-hex and api-dex-hex) showed almost no increase. Xiaoyan96 was likely the origin of the high-flavonoid phenotype in Xiaoyan6hao and its progeny XY22 (Figure 3c). Yanshi4hao and Zhengmai16 were excellent high flavonoids, and their HF trait was inhabited in BonongAikang58 (BNAK58), but was maintained in Zhengmai22 (Figure 3c). BNAK58 was famous for its high yield and resilience to multiple pathogens. Moreover, the cultivars with middle or high powdery mildew resistance tended to contain higher flavonoids (Figure 3d).

### 3.4. Flavonoids Protect Wheat from Powdery Mildew in an EMS Mutant Library

There was a positive correlation between flavonoid content and powdery resistance in MCC, however this correlation could be attributed to the presence introducing disease-resistant genes in MCC. To further elucidate the correlation between the content of flavonoids and powdery mildew resistance in a relatively straightforward genetic background, we investigated the growth of powdery mildew in a tetraploid wheat EMS mutant library, focusing on leaf-increased flavonoid (*lif*) mutants and leaf-reduced flavonoid (*lrf*) mutants [5].

**Figure 4:**
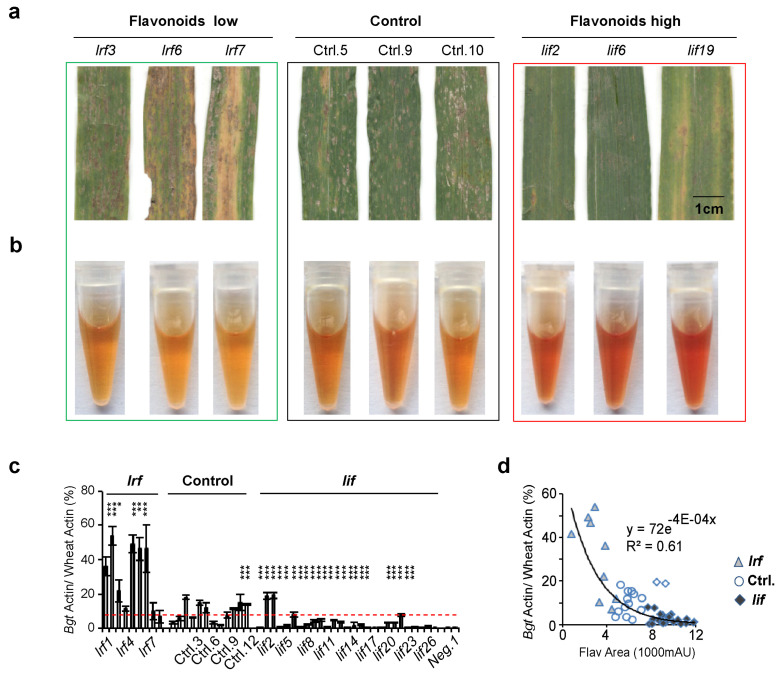
Powdery mildew growth in durum wheat flavonoid mutants in the field. (**a**) Powdery mildew growth in flavonoid mutants and controls in the field. (**b**) Colorimetric reactions of flavonoids in the corresponding leaves. Note that the colorimetric reaction of flavonoids produced the red color. (**c**) Quantification of powdery mildew DNA in flavonoid mutants and controls. Two samples without powdery mildew infection were negative controls (Neg.). Student’s *t*-test value, * *p* < 0.05, ** *p* < 0.01, *** *p* < 0.001. *n* = 4. (**d**) Correlation between the content of powdery mildew and flavonoid total peak areas in the HPLC analysis.

In field trials, less powdery mildew was observed in over 80% of the *lif* mutants. In comparison, 2/3 of the *lrf* mutants suffered more severe powdery mildew infections (Figure 4a, Supplementary Figure S1). The changes in flavonoids in *Bgt*-infected leaves were further validated by the colorimetric reaction (Figure 4b), indicating that the highly accumulated flavonoids could very likely protect wheat from powdery mildew. A negative exponential correlation existed between the total flavonoid peak areas and powdery mildew DNA ratios amongst these samples (Figure 4c).

**Figure 5:**
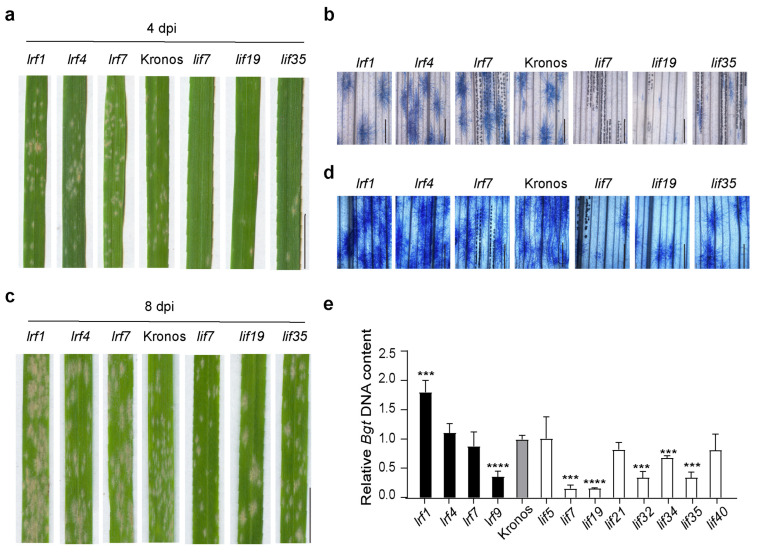
Powdery mildew growth in durum wheat flavonoid mutants in a controlled environment. (**a**) A representative picture of powdery mildew symptoms on the leaf surface is at four dpi. (**b**) Staining of *Bgt* hyphae in the leaf at four dpi. (**c**) A representative picture of powdery mildew on the leaf surface at eight dpi. (**d**) Staining of *Bgt* hyphae in the leaf at eight dpi. (**e**) Quantification of *Bgt* biomass at 8 dpi. *n* = 4. Student’s *t*-test value, *** *p* < 0.001, **** *p* < 0.0001.

To further verify the protective function of flavonoids against powdery mildew in a controlled environment, we inoculated the young plants with a widely used *Bgt* isolate E09 by spreading the pathogen on seedling leaves. The pathogen grew well in the control and the *lrf* samples with lower flavonoids, but fewer pustules appeared in the *lif* samples (Figure 5a). At four days post-inoculation (dpi), in six out of the eight *lif* samples the pathogen germinated. Still, the areas of hyphae were~20% smaller than that of *lrf* and control (Figure 5b).

We further checked the effect of flavonoids on powdery mildew at a later stage. At eight dpi, fewer powdery mildew pustules were formed in the *lif* samples than in the WT control (Figure 5c). However, the fungal hyphae fully developed in all samples (Figure 5d). In the *lif* samples, five out of the eight showed very significant reductions (*p* < 0.001) in the fungal DNA (Figure 5e), indicating reductions in pathogen biomass. The above results strongly supported flavonoid’s protective role against powdery mildew on wheat.

## 4. Discussion

This study investigated the trend in flavonoids in Chinese cultivars, landraces, and some international parental lines. In agreement with the flavonoid variations, these high-flavonoid cultivars have significantly higher antioxidant potential. Therefore, high-flavonoid cultivars are excellent genetic resources for boosting the health-promoting functions of wheatgrass. Furthermore, this study showed the main flavonoids in wheatgrass as C-glycosylated flavonoid glycosides, with the aglycones predominantly being apigenin, luteolin, and chrysoeriol. Glycosylation modifications include not only monosaccharide glycosylation, but also disaccharide and bis-glycosylation. As glycosylation modifications can potentially change the biochemical characters of flavonoids, cultivars with different combinations of flavonoid core and glycosylation modifications could meet the specialized needs of different customers. Although there were no commercial standards for each flavonoid glycoside present in wheatgrass, this work paved a critical step to correlate the flavonoids with antioxidant activities and potential anti-microbial functions.

The current study explored a collection of 145 wheat varieties, which provided insights into the content of flavonoids in wheat varieties from different historical periods in China. First, this study revealed that some cultivars introduced to China 60 years ago were rich in flavonoids, such as Mexipak 66 (Figure 2). Mexipak 66 was first introduced to India and Pakistan and also has significant contributions to the Chinese wheat-breeding history. The current experiment suggested that Mexipak 66 could contribute to the high flavonoids in MCC. Second, this work found that wheat varieties developed in the past 20 years have a higher content of flavonoids, whereas few earlier breeders studied flavonoid content. This involuntarily increase is consistent with the correlation between flavonoids and resilience to environmental stresses in plants. The observation in this work provides a potential direction for subsequent selection and breeding of wheat elite cultivars that show advantages in their resilience to climate change.

Genetic factors play a significant role in the accumulation of flavonoids [17]. Following our discovery that the content of flavonoids in the wheatgrass of Chinese wheat varieties has increased with the breeding process, we hypothesized that this may be due to the accumulation of flavonoids in some founder parents, leading to a continuous accumulation in subsequent varieties. Indeed, we found that the flavonoid content in some founder parents, such as XiaoYan96 and YanShi4Hao, is higher than in other varieties from the same era. However, there are also essential founder parents with low flavonoid content, such as YuMai2 and AiFeng3 (Supplementary Figure S4). XiaoYan96 is famous for its high yield potential and resilience to fungal diseases, and the enriched flavonoids could contribute to disease tolerance in XiaoYan96 and its derived cultivars.

Flavonoids conferred antimicrobial activity widely in plants, e.g., desert cotton (*Avera Javanica*), flax (*Linum ustitatissimum*), and alfalfa (*Medicago sativa* L.) [18,19]. The flavonoid mutants used here provided novel information for the role of different flavonoids in wheat fungal resistance. The correlation analysis between individual flavonoids and *Bgt* resistance revealed four Luteolin C-glucoside derivatives with dominant contributions. The above phenomenon was consistent with a previous report that luteolin had a more potent inhibition on *Colletotrichum sublineolum* spore germination in vitro than apigenin [20]. The current data suggest a weak positive correlation between luteolin derivatives and *Bgt* resistance in MCC. We studied a tetraploid wheat EMS mutant population to minimize the effect of unknown *Bgt* resistance genes. The luteolin glucosides, based on the reduction in plaque coverages in *lif* mutants at the early stage, could negatively regulate the germination of the *Bgt* spore. As a result, luteolin C-glucoside is promising to select potentially resistant plants and improve fungal resistance in future wheat breeding programs.

Despite the positive correlation between flavonoids and *Bgt* tolerance in cultivars (Figure 3) and EMS mutants (Figure 4 and Figure 5), stronger genetic evidence is still needed to support flavonoids’ protective role in wheat against fungal pathogens. The cloning of candidate genes underpinning flavonoid accumulation and transgenic plants will significantly benefit the explanation of why recently grown varieties have accumulated more flavonoids. The correlation of flavonoids with wheat resistance could be a clue. Identifying critical genes in the cultivars and EMS mutants and studying their working mechanisms underlying the accumulation of flavonoids in wheatgrass may answer this question [21]. We are now constructing subsequent genetic materials to clone the genes and elucidate the mechanisms underlying flavonoid variations.

## 5. Conclusions

In summary, our study on flavonoids in the wheatgrass of significant wheat varieties from different periods in China identified several wheat varieties with high flavonoid content and strong antioxidant properties, but also revealed a phenomenon of the accumulation of flavonoids in Chinese wheat varieties throughout the breeding process. This discovery paves the way for the accelerated application of high-nutrition wheatgrass. It lays an essential foundation for future research on breeding wheat with high flavonoid content and the mechanisms behind flavonoid accumulation.

## Figures and Tables

**Table 1:** Mobile phase elution method.

Time/min	Acetonitrile/%	ddH_2_O/%
0	15.0	85.0
8	18.0	72.0
13	30.0	70.0
19	45.0	55.0
20	100.0	0.0
21	100.0	0.0
22	15.0	85.0
23	15.0	85.0

## Data Availability

The raw data supporting the conclusions of this article is included in the Supplementary Materials.

## Supplementary Materials

The following supporting information can be downloaded at: https://www.mdpi.com/article/10.3390/antiox13080899/s1, Figure S1. Analysis of Genomic Resequencing Data from 145 Wheat Samples; Figure S2. UHPLC-DAD chromatograms of flavonoids in wheatgrass; Figure S3. Principal component analysis (PCA) of the composition of flavonoids in wheatgrass; Figure S4. The phenotype of powdery mildew in field; Table S1. The origin and breeding time of the cultivars studied in this work; Table S2. The contents of flavonoids and anti-oxidant potentials of the cultivars studied in this work.
