# Supplementary material for: Change of Flavonoid Content in Wheatgrass in a Historic Collection of Wheat Cultivars
Source: Antioxidants (Basel). 2024 Jul 25;13(8):899. doi: 10.3390/antiox13080899 (PMC11351879; doi:10.3390/antiox13080899)
Supplement: Supplementary file 1 [file antioxidants-13-00899-s001.zip › antioxidants-3061985-supplementary.pdf]

Supplementary Materials:

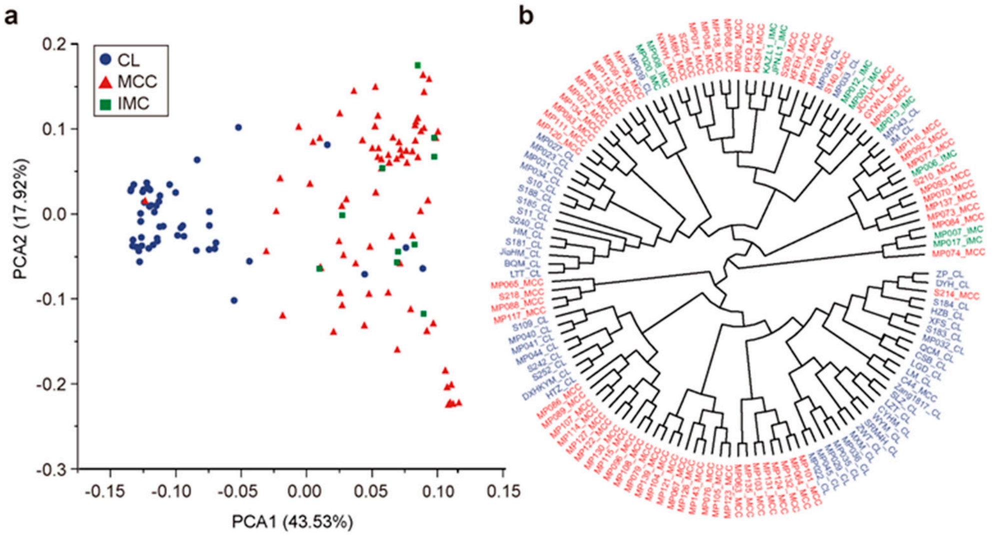

Figure S1. Analysis of Genomic Resequencing Data from 145 Wheat Samples. **(a) A Principal Component Analysis (PCA) on the resequencing data of the genomes to observe differences in their genetic backgrounds. The first principal component accounts for 43.5% of the contribution, and the second principal component accounts for 17.92%. Blue circles represent samples from the CL group, red triangles represent samples from the MCC group, and green squares represent samples from the IMC group. (b) An evolutionary tree for the 145 samples was constructed based on SNP site information.**

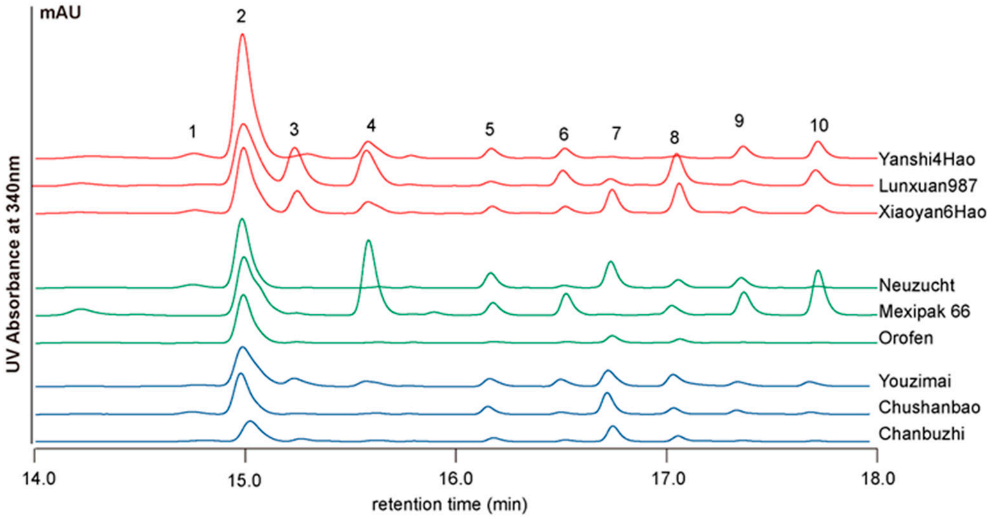

Figure S2. UHPLC-DAD chromatograms of flavonoids in wheatgrass. **The chromatograms show the profiles from the retention time of 14 to 18 minutes, with blue representing samples from the CL group, red representing samples from the MCC group, and green representing samples from the IMC group.**

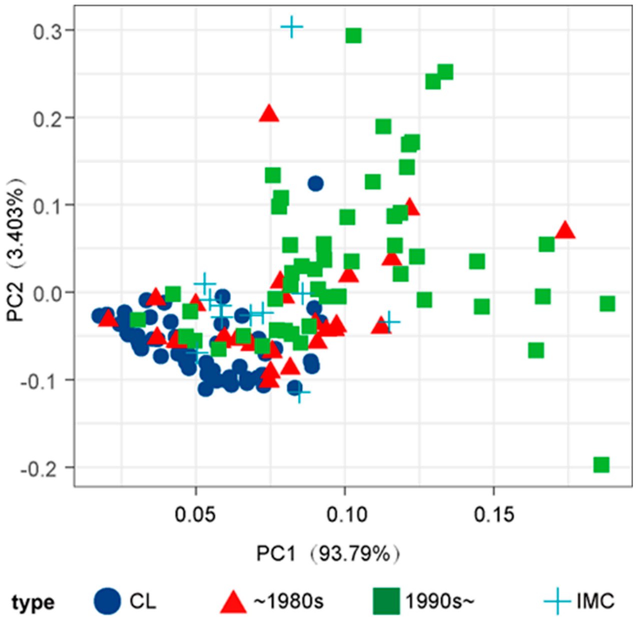

Figure S3. Principal component analysis (PCA) of the composition of flavonoids in wheatgrass. **The materials from the MCC group were divided into the following two subgroups: the "Pre-1980s" group, which includes materials bred up to and including the 1980s; and the "Post-1990s" group, which includes materials bred from the 1990s onwards.**

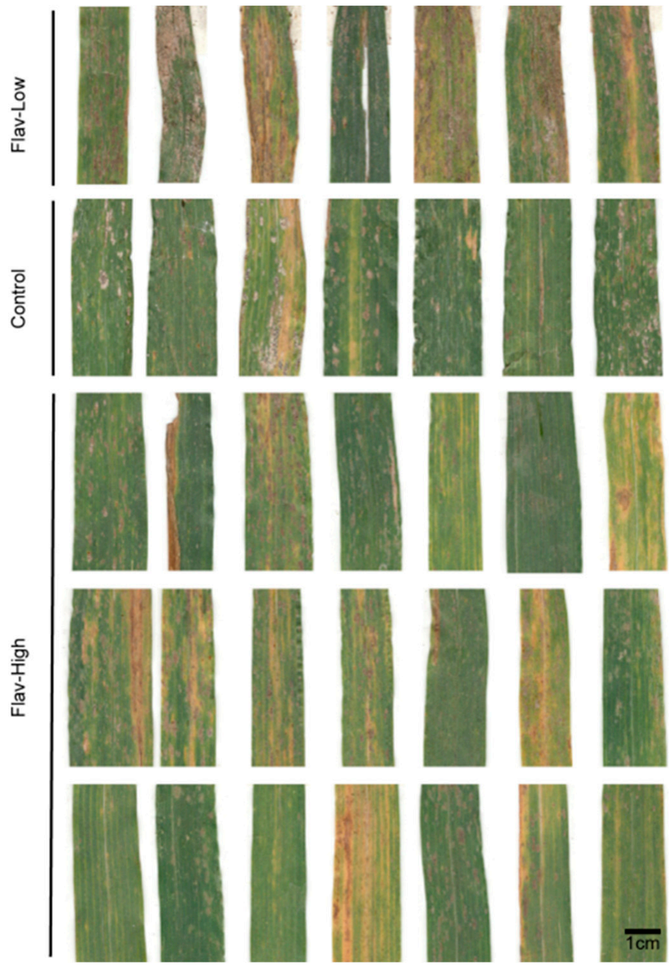

Figure S4. The phenotype of powdery mildew in the field.

Table S1. The origin and breeding time of the cultivars studied in this work.

Table S2. The content of flavonoids in, and the antioxidant potentials of, the cultivars studied in this work.
